# 98 Care Discontinuity in Burn Patients: Does it Impact Long-Term Outcomes?

**DOI:** 10.1093/jbcr/irad045.071

**Published:** 2023-05-15

**Authors:** Manuel Castillo-Angeles, Lauren J Shepler, Caitlin Orton, Colleen Ryan, Jeffrey Schneider, Anupama Mehta

**Affiliations:** Brigham and Women's Hospital, Boston, Massachusetts; Spaulding Rehabilitation Hospital, Harvard Medical School, Boston, Massachusetts; University of Washington, Seattle, Washington; Massachusetts General Hospital, Boston, Massachusetts; Spaulding Rehabilitation Hospital/Harvard Medical School/Rehabilitation Outcomes Center at Spaulding, Boston, Massachusetts; Brigham and Women's Hospital, Boston, Massachusetts; Brigham and Women's Hospital, Boston, Massachusetts; Spaulding Rehabilitation Hospital, Harvard Medical School, Boston, Massachusetts; University of Washington, Seattle, Washington; Massachusetts General Hospital, Boston, Massachusetts; Spaulding Rehabilitation Hospital/Harvard Medical School/Rehabilitation Outcomes Center at Spaulding, Boston, Massachusetts; Brigham and Women's Hospital, Boston, Massachusetts; Brigham and Women's Hospital, Boston, Massachusetts; Spaulding Rehabilitation Hospital, Harvard Medical School, Boston, Massachusetts; University of Washington, Seattle, Washington; Massachusetts General Hospital, Boston, Massachusetts; Spaulding Rehabilitation Hospital/Harvard Medical School/Rehabilitation Outcomes Center at Spaulding, Boston, Massachusetts; Brigham and Women's Hospital, Boston, Massachusetts; Brigham and Women's Hospital, Boston, Massachusetts; Spaulding Rehabilitation Hospital, Harvard Medical School, Boston, Massachusetts; University of Washington, Seattle, Washington; Massachusetts General Hospital, Boston, Massachusetts; Spaulding Rehabilitation Hospital/Harvard Medical School/Rehabilitation Outcomes Center at Spaulding, Boston, Massachusetts; Brigham and Women's Hospital, Boston, Massachusetts; Brigham and Women's Hospital, Boston, Massachusetts; Spaulding Rehabilitation Hospital, Harvard Medical School, Boston, Massachusetts; University of Washington, Seattle, Washington; Massachusetts General Hospital, Boston, Massachusetts; Spaulding Rehabilitation Hospital/Harvard Medical School/Rehabilitation Outcomes Center at Spaulding, Boston, Massachusetts; Brigham and Women's Hospital, Boston, Massachusetts; Brigham and Women's Hospital, Boston, Massachusetts; Spaulding Rehabilitation Hospital, Harvard Medical School, Boston, Massachusetts; University of Washington, Seattle, Washington; Massachusetts General Hospital, Boston, Massachusetts; Spaulding Rehabilitation Hospital/Harvard Medical School/Rehabilitation Outcomes Center at Spaulding, Boston, Massachusetts; Brigham and Women's Hospital, Boston, Massachusetts

## Abstract

**Introduction:**

Care discontinuity, or the care that is provided by disparate healthcare professionals when patients are readmitted to a hospital different than their original admission (non-index hospital), has been associated with worse outcomes among surgical patients. However, little is known about its effect on burn patients. Our aim was to determine the impact of care discontinuity on long-term outcomes in a national sample of burn patients.

**Methods:**

The longitudinal Burn Model System National Database was queried from 1996 to 2021. All adult burn patients from participating sites were included. Care discontinuity was defined by having therapy or burn-related surgeries at non-index hospitals. The main outcomes were the physical (PCS) and mental (MCS) health component summary scores of the Veterans RAND 12 (VR-12) score at 6 and 12 months after injury. Multivariable regression was used to examine the association between care discontinuity and long-term outcomes, adjusting for patient characteristics and burn injury severity.

**Results:**

A total of 1,866 burn patients were included. Mean age was 43.36 (SD 15.85) years, 73% were male and 76% were white. Mean PCS scores were 43.56 (SD 10.95) and 45.17 (SD 11.18); and mean MCS scores were 48.00 (SD 12.60) and 48.25 (SD 12.55) at 6 and 12 months, respectively. Care discontinuity was reported in 423 (22.6%) burn patients, who reported having therapy or burn-related surgeries at a non-index hospital. In adjusted analyses, care discontinuity was associated with worse PCS at both 6 months (Coefficient -2.18, 95% Confidence Interval [CI] -3.44 – -0.91, p=0.001), and 12 months (Coefficient -2.16, 95% CI -3.50 – -0.82, p=0.002), compared to patients having therapy/surgery at index hospitals. Care discontinuity was not associated with MCS scores at 6 (Coefficient 0.54, 95% CI -0.04 – 2.03, p=0.473) and 12 months (Coefficient -0.90, 95% CI -2.49 – -0.67, p=0.262).

**Conclusions:**

Care discontinuity was significantly associated with lower physical health component scores at long-term follow up, but not with mental health component scores. Future studies should focus on developing strategies to ensure continuity of care and/or identifying out of system patients to provide targeted resources.

**Applicability of Research to Practice:**

Understanding the implications of care fragmentation on longitudinal outcomes may help clinicians develop strategies to ensure care continuity and consequently better outcomes.

